# The association of anxiety and other clinical features with CACNA1C rs1006737 in patients with depression

**DOI:** 10.1515/tnsci-2022-0244

**Published:** 2022-09-24

**Authors:** Henrik Dam, Jens O. D. Buch, Annelaura B. Nielsen, Pia Weikop, Martin B. Jørgensen

**Affiliations:** Mental Health Center Copenhagen, University Hospital of Copenhagen, Edel Sauntes Alle 10, 2100 Copenhagen O, Denmark; Novo Nordisk Foundation Center for Protein Research, University of Copenhagen, Copenhagen, Denmark; Center for Translational Neuroscience, University of Copenhagen, Copenhagen, Denmark

**Keywords:** CACNA1C, rs1006737, anxiety, depression, ECT, first admission

## Abstract

**Background:**

The CACNA1C protein is a L-type calcium channel, which influence affective disorders.

**Purpose:**

The purpose of the present study was to examine the possible association between the different genotypes of rs100677 CACNA1C gene and anxiety and other clinical symptoms in patients with unipolar depression.

**Patients and controls:**

A total of 754 patients and 708 controls from the Danish Psychiatric Biobank participated.

**Results:**

A significant correlation was found between anxiety and the A allele. It was further found that patients with the A allele more often were treated with electroconvulsive therapy and patients with the AA phenotype had the highest age.

**Limitations:**

The only information about controls was their sex and that they were recruited from the blood bank. Two types of inclusion criteria were used. The clinical data were not complete for all patients.

## 1 Introduction

Major depressive disorder (MDD) is one of the most frequent and severe psychiatric conditions, with an estimated prevalence reaching 15% in the general population [1]. MDD is twice as prevalent in women compared to men [2]. It is a painful illness and much of the suffering is due to anxiety, which nearly 50% of the patients experience. Most fatal is the suicidium, which often is executed by violent methods, e.g., traffic accidents, where it is estimated that above 2 % of the traffic accidents are suicide behaviors [3]. Both depression and anxiety are multifactorial diseases with psychosocial and biological etiology. For example, the recent corona lockdown in Italy causes psychological symptoms in fragile individuals [4,5].

It is well established that polymorphisms in genes coding for *L*-type calcium channels influence affective disorders especially bipolar disorders [6]. Of those, the rs1006737 polymorphism within CACNA1C is perhaps the best studied and most influential in MDD, but at the diagnostic level, large samples are required for association studies [7]. A recent reappraisal [8] argues that voltage-gated calcium channels may be a potential therapeutic target in psychiatry.

Neuroinflammation can be a factor in developing depression and several possible mechanisms have been proposed [9]. One of the mechanisms include voltage-gated calcium channels [10].

In patients with major depression, more than 50% experience anxiety [11] and in search for endophenotype studies on rs1006737, higher levels of anxiety were found among healthy individuals carrying the A allele compared to the G allele [12]. Two other studies also find association between the A allele in rs1006737 and anxiety symptoms in healthy persons [13,14]. A study [15] found a differential effect of the SNP on emotional lability between men and women in the general population.

Two meta-analyses find association between the A allele in rs1006737 and depression [7,16]. In the STAR*D study on depressed patients, rs1006737 SNP was associated with a protective effect on baseline depression severity and insomnia and agitation, but an increased risk of treatment-emergent suicidality [17].

We aimed to employ data and samples from patients with MDD from the Danish Psychiatric Biobank to determine associations between the rs1006737 SNP and clinical features.

The abovementioned studies indicate that patients with the A allele seem more prone to develop psychological symptoms. Furthermore, that pregabalin, an anxiolytic drug, is a ligand for the voltage dependent calcium channel. This led to the main hypothesis for this study that depressive patients with the A allele in rs1006737 are more prone to develop anxiety.

The primary purpose was to evaluate a possible association between anxiety and the rs1006737 polymorphism in the CACNA1C gene in depressive patients. The secondary purpose was to evaluate a possible association between the rs1006737 polymorphism and (1) severity of illness and (2) alcoholism in depressive patients.

## 2 Methods

### 2.1 Design

The patients were recruited from hospital departments where a high degree of psychopathology can be expected and the patients’ disease history will be well described from the treatment course.

The clinical data were collected in a standardized manner with a protocol including: age, sex, ethnicity, International Classification of Diseases version 10 (ICD-10) diagnoses, psychopathology including anxiety at admission, psychiatric illness in family, age for first psychiatric admission, age for first psychopharmacological treatment, number of admissions, suicide attempts, use of alcohol and drugs through life, treatment with lithium or electroconvulsive therapy (ECT) through life, and trauma in childhood. This information was obtained by a interview and/or extracted from patients’ file.

### 2.2 Patients and controls

Patients were selected from the Danish Psychiatric Biobank based on the diagnosis of unipolar depression according to the ICD-10 diagnostic criteria. The Danish Psychiatric Biobank is a collaborative effort between psychiatric departments in Copenhagen to collect blood samples from psychiatric patients from 2002 to 2015.

The majority (85%) of the patients were ethnical Danish, that is, the patients and both parents were born in Denmark, while in a minor fraction of the cases (15%), one parent was Caucasian and born outside Denmark in another North-western European country, primarily Sweden or Norway, secondarily in Germany, The Netherlands, England, or France [18].

Patients were selected from the Danish Psychiatric Biobank if they were given a clinical diagnosis of unipolar depression according to the ICD-10 when they were admitted to the psychiatric department. The ICD-10 diagnosis was later confirmed either 1) by a structured interview with “Scheduled Clinical Assessment Neuropsychiatry” by a trained research psychiatrist or 2) the diagnoses were approved from the file by a trained research psychiatrist or 3) the patient had been admitted at least 2 times with a clinical diagnosis of depression according to the ICD-10. A total of 754 patients participated (241 males and 513 females). The clinical information was obtained by interview with the patients or extracted from the patient’s file.


**Ethical approval:** The research related to human use has been complied with all the relevant national regulations, institutional policies and in accordance the tenets of the Helsinki Declaration, and has been approved by the Danish Scientific Committees (J.nr. 01-024/01) and the Danish Data Protection Agency.
**Informed consent:** Informed consent has been obtained from all individuals included in this study.

### 2.3 Controls

Controls were blood donors from the blood bank on Rigshospitalet in Copenhagen. To be a blood donor, the person had to be between 17 and 60 years of age, healthy, and with a weight of at least 50 kg. Blood donor samples were obtained according to the same protocol that was used to recruit patients [18]. The controls were anonymous and only the sex was known. Seven hundred and eight controls participated (272 males and 436 females).

### 2.4 Anxiety

The presence of anxiety was assessed in two ways.

It was recorded from the patients file whether there had been described anxiety or not in the first week after admission.

If the patient scored 2 or more on item 10 (feeling anxious) on Hamilton 17 Rating Scale [19]. The evaluation could be performed at any time under an admission or treatment in an outpatient clinic.

### 2.5 Alcohol abuse

It was recorded from the patient file if the patient had a diagnosis of alcohol abuse, had been in treatment with disulfiram, had been in treatment in an out-patients clinic for alcoholics, or if there was other information indicating abuse of alcohol.

### 2.6 Suicidality

It was extracted from the patient file on a 5-item scale ranging from no suicidal thoughts (0), suicidal thoughts (1), one nonserious suicidal attempt (2), more than one nonserious suicidal attempt (3), one serious suicidal attempt (4), and more than one serious suicidal attempt (5).

### 2.7 Treatment

It was recorded if the patients had been in treatment with ECT at any time in their life.

### 2.8 Start of treatment

Age of first medication and age at first admission were obtained by interview or extracted from the patient’s file.

### 2.9 Analysis

**Figure 1:**
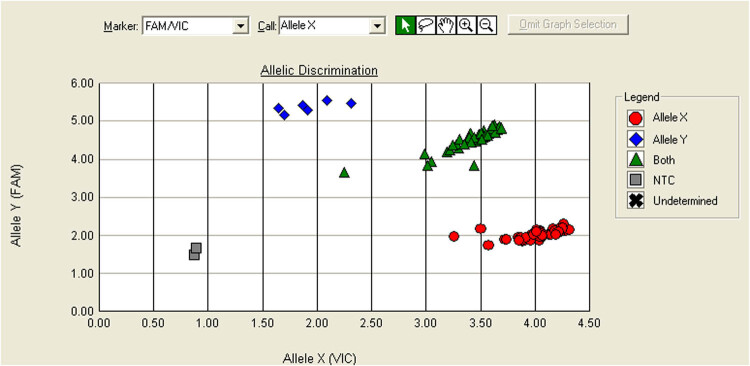
Allelic discrimination plot. The *X*-axis is the VIC^®^ fluorescent intensity, and the *Y*-axis is the FAM™ fluorescent intensity. Samples showing high intensity on only one axis (relative to blank samples, seen in gray) are homozygous for either the A allele (VIC^®^) or the G allele (FAM™), highlighted here in red or blue. Samples showing both VIC^®^ and FAM™ fluorescence intensities are heterozygous, highlighted here in green.

We used TaqMan^®^ SNP Genotyping Assay (Thermo Fisher Scientific, Waltham, MA, USA), a standard method for detecting single-nucleotide polymorphisms, to determine CACNA1C (A/G) genotype. Two labeled oligonucleotide probes are used to identify each allelic variant; in this study, the flurophoresVIC^®^ (4,7,2-trichloro-7-phenyl-6-carboxylfluorescein) and FAM™ (6-carboxy-fluorescein) reported the A/G SNP. The oligonucleotide probes are complementary to the sequence of interest, labeled at the 5′ end with a reporter dye and at the 3′ end with a quencher dye. The probe anneals to the DNA sequence of interest and during PCR amplification, the 5′–3′ exonuclease activity of the DNA TaqMan^®^ polymerase degrades the probe, separating the reporter and the quencher dye resulting in a fluorescent signal, increasing for each amplification round. The proximity between reporter and quencher in unbound probes will block the signal. The fluorescent intensity was measured by the Applied Biosystems^®^7500 Fast Real-Time PCR System (Thermo Fisher Scientific) with fluorescent emission at 554–530 nm for VIC^®^ and FAM™. In the allelic discrimination analysis, the quantitative contribution of each dye for each sample was used to produce a scatterplot, where signal for FAM™ or VIC^®^ exclusively indicates homozygous samples and increased signal for both dyes indicates heterozygosity (Figure 1). Non-template controls were included to calibrate the scatterplot, and triplicates of each sample were used to ensure confident allelic determination. 5.4% of the sample failed the analysis. The Hardy–Weinberg equation was fulfilled.

### 2.10 Statistics

Statistics was performed with SPSS 18. To compare frequencies, the chi square test was used. The analysis of variance (ANOVA) was used to compare means and the Bonferroni correction was used to compensate for multiple testing. Tests were performed for eight factors: depression, anxiety ×2, onset ×2, suicidality, alcohol, and ECT. A *p*-value of 0.006 was considered statistically significant.

## 3 Results

No difference in distribution of genotypes was found between patients with unipolar depression and controls (Table 1), neither without nor with division of the population according to sex. Odds ratio for the A allele was found to be 1.04 (95% CI: 0.94–1.10).

**Table 1:** Distribution of genotypes in patients with unipolar depression and controls

Genotype	Depressed, *N* (%)	Controls, *N* (%)
GG	330 (44.0)	319 (45.1)
AG	333 (44.4)	309 (43.7)
AA	87 (11.6)	79 (11.2)

### 3.1 Anxiety

#### 3.1.1 At admission

No difference in frequency was found between genotypes in patients with or without anxiety (chi square, *p* = 0.061) (Table 2). A significant difference was found between genotypes of patients with anxiety and controls (chi square, *p* = 0.011) (Table 3).

**Table 2:** Distribution of genotypes in patients with and without anxiety at admission

	Anxiety	No anxiety
GG	51	69
AG	88	67
AA	18	16

**Table 3:** Distribution of genotypes in patients with anxiety at admission and controls

	Pt. with anxiety	Controls
AA	18	79
AG	88	309
GG	51	319

A significant difference was found for the A allele (AA + AG/GG) between patients with and without anxiety (chi square, *p* = 0.013) (Table 4) with the ODs ratio for the A allele of 1.45 (1.04–2.03).

**Table 4:** Distribution of genotypes in patients with and without depression at admission

	Anxiety	No anxiety
AA + AG	106	83
GG	51	69

A significant difference for the A allele was also found between patients with anxiety and controls (chi square, *p* = 0.0036).

**Figure 2:**
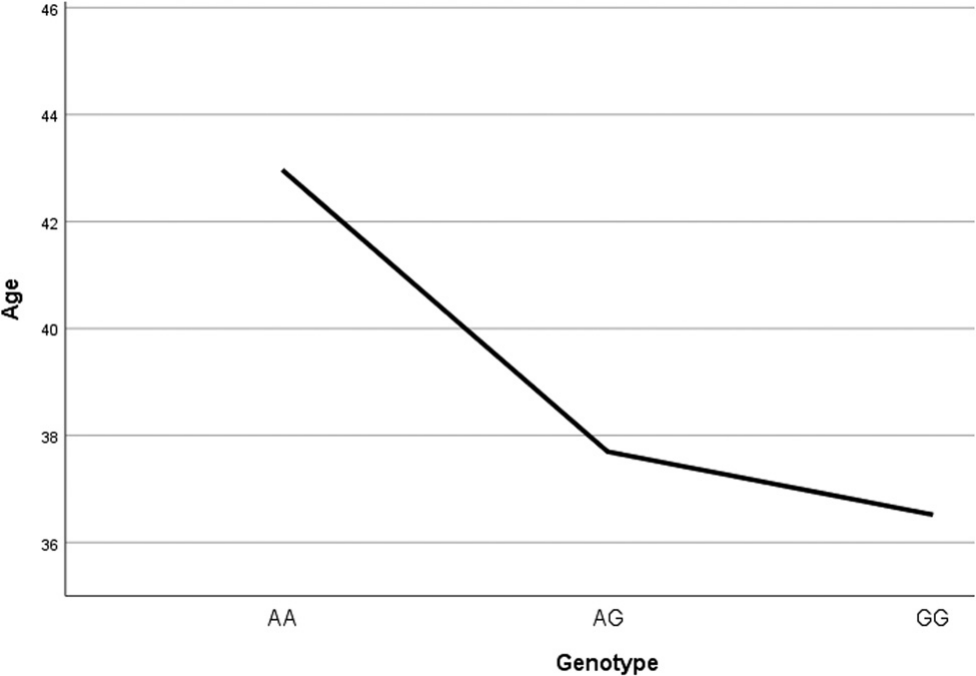
Age at first medication according to genotype.

When the patients were divided according to sex, the frequency of anxiety was significantly higher in females (121 anx/92 non-anx) than in males (36 anx/62 non-anx) (*p* = 0.001). A significant difference was found for the A allele between woman with and without anxiety (chi square, *p* = 0.014) (Figure 2). No significant difference was found for the A allele between males with and without anxiety (chi square, *p* = 0.59).

#### 3.1.2 By Hamilton item 10

In 397 patients, the HDRS evaluation was made (136 males, 261 females, mean 11.5, median 11, SD 7.8, min 0, max 55). No significant difference in frequency of anxiety was found between the genotypes or for the A allele between patients with anxiety or not. For women, a trend toward increased anxiety was found for the A allele (*p* = 0.079 two-sided test).

### 3.2 Onset of illness

**Figure 3:**
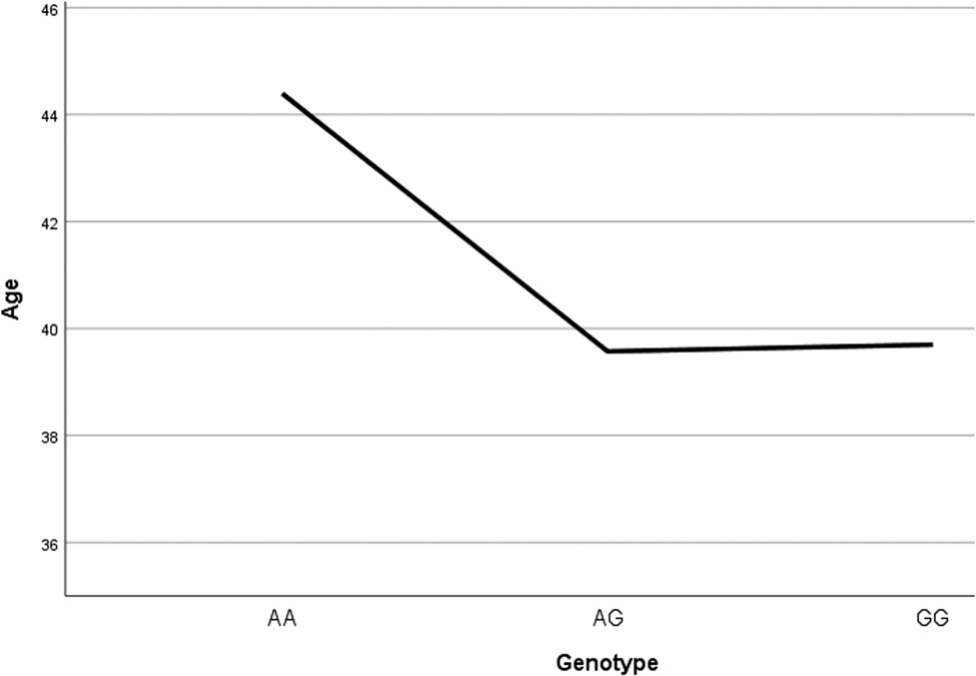
Age at first admission according to genotype.

**Figure 4:**
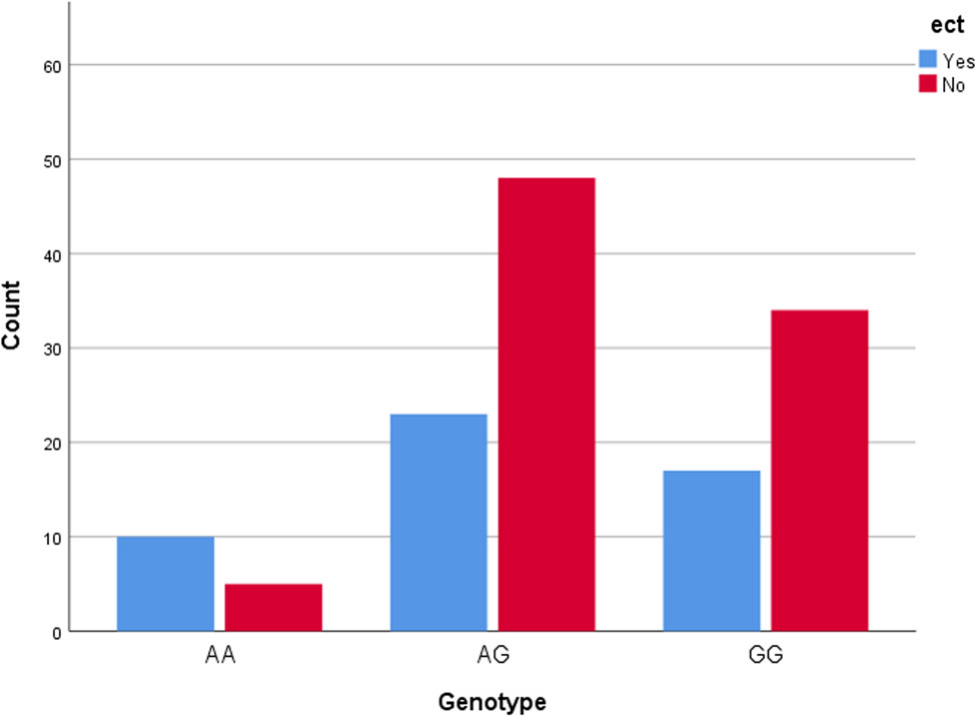
ECT according to genotype.

In this analysis, the age of first treatment and the age at first admission was taken as sign of start of illness. It is seen that, for both, the AA genotype had the highest age of incidence (Figures 2 and 3, Tables 5 and 6). By the ANOVA test, this reached significance for the age of first treatment (*p* = 0.010) but not for the age at first admission (*p* = 0.329) (Figure 4).

**Table 5:** Age at first medication according to genotype

Genotype	Number	Mean	SD
GG	224	36.5	13.9
AG	244	37.7	14.7
AA	56	43.0	15.0

**Table 6:** Age at first admission according to genotype

Genotype	Number	Mean	SD
GG	86	39.7	14.7
AG	103	39.6	14.2
AA	23	44.4	14.8

### 3.3 Suicidal attempt

A total of 107 patients had performed a suicidal attempt and 357 had not. No significant difference in the frequency of suicidal attempts was found between the genotypes according to the chi square test (*p* = 0.356).

### 3.4 ECT

Fifty patients had received ECT and 87 had not. There was a significant difference in the frequency of ECT between the genotypes (*p* = 0.036) (Table 7). The OD ratio for the A allele was 1.51 (0.91–2.50).

**Table 7:** ECT according to genotype

Genotype	+ ECT	–ECT
GG	17	34
AG	23	46
AA	10	5

### 3.5 Alcohol

A total of 104 patients had an abuse of alcohol and 360 did not have an abuse of alcohol. No difference in frequency of alcohol abuse was found between the different genotypes (*p* = 0.425).

## 4 Discussion

The results of the present cohort support the hypothesis of a sex-specific influence of the SNP rs1006737 within CACNA1C on traits associated with emotional lability and resilience in the general population [15]

A trend toward correlation was found between frequency of anxiety as measured by HDRS 17, item 10, and the genotypes, but it did not rich significance.

Secondary analyses included: start of illness, suicide, alcohol abuse, and ECT. In the secondary analyses, patients with the AA genotype showed highest age for start of treatment (*p* = 0.01) and admission to hospital (ns). This was not found in another study [20], but it was found that the C allele in rs1034936 in the CACNA1C gene was associated with a higher age for start of illness

Patients with the A allele showed the highest frequency of ECT treatment (*p* = 0.036). Perhaps this finding of increased anxiety and increased age reflect a phenotype perceived by clinicians as particularly suited for ECT treatment. ECT is considered increasingly effective with increasing age [21]

No significant difference in the frequency of suicidal attempts was found between the genotypes (*p* = 0.356). This is in accordance with a study in bipolar patients [22].

In the present context, we conceived alcohol dependency as a proxy for anxiety [23] but no correlation was found between genotype and alcohol abuse and no information could be found concerning alcohol abuse and the rs1006737 polymorphism in the literature. Another polymorphism (rs1034936) in the CACNA1C gene appears to correlate with alcohol abuse in bipolar patients, [24].

No association was found between the different genotypes and the diagnosis of unipolar depression compared to controls. This is in accordance with one earlier study [25] whereas another study found an association [26].

In conclusion, this study finds that the A allele in r1006737 may be a risk allele for psychological vulnerability. Woman with the A allele are more prone to develop anxiety. The patients with the A allele had a trend toward earlier initiation of psychopharmacological treatment and were most often treated with ECT.

This can in the future be used as a marker of psychological vulnerability in projects selecting people for the prevention of psychiatric illness.

Mothering’s in voltage-gated calcium channels may carry a synergetic effect.

It can be relevant to study the rs1006737 polymorphism in other disorders with anxiety. This could be, e.g., panic disorder and generalized anxiety. It could also be relevant to study this polymorphism in conditions with more subtle anxiety as avoidant personality disorder.

### 4.1 Limitations

The only information about controls were their sex and that they were recruited from the bloodbank.

The clinical data were not complete for all patients. The patients were recruited from different hospitals and it was not possible to get access to all patient’s files.

The ICD-10 inclusion diagnosis was later confirmed in one of the three different ways: either by a structured interview Scheduled Clinical Assessment Neuropsychiatry by a trained research psychiatrist, the diagnoses were approved from the file by a trained research psychiatrist, or the patient had been admitted at least two times with a clinical diagnosis of depression according to the ICD-10.

## References

[1] Andrade L, Caraveo-Anduaga JJ, Berglund P, Bijl RV, De Graaf R, Vollebergh W, et al. The epidemiology of major depressive episodes: Results from the international consortium of psychiatric epidemiology (ICPE) surveys. Int J Methods Psychiatr Res. 2003;12(1):3–21. 10.1002/mpr.138 PMC6878531 12830306

[2] Anker JJ, Kushner MG. Co-occurring alcohol use disorder and anxiety: Bridging psychiatric, psychological, and neurobiological perspectives. Alcohol Res. 2019;40(1). 10.35946/arcr.v40.1.03 PMC6927748 31886106

[3] Calabro M, Mandelli L, Crisafulli C, Lee SJ, Jun TY, Wang SM, et al. Genes involved in neurodevelopment, neuroplasticity and major depression: No association for CACNA1C, CHRNA7 and MAPK1. Clin Psychopharmacol Neurosci. 2019;17(3):364–8. 10.9758/cpn.2019.17.3.364 PMC6705106 31352702

[4] Casamassima F, Huang J, Fava M, Sachs GS, Smoller JW, Cassano GB, et al. Phenotypic effects of a bipolar liability gene among individuals with major depressive disorder. Am J Med Genet B Neuropsychiatr Genet. 2010;153B(1):303–9. 10.1002/ajmg.b.30962 19388002

[5] Cipriani A, Saunders K, Attenburrow MJ, Stefaniak J, Panchal P, Stockton S, et al. A systematic review of calcium channel antagonists in bipolar disorder and some considerations for their future development. Mol Psychiatry. 2016;21(10):1324–32. 10.1038/mp.2016.86 PMC5030455 27240535

[6] Erk S, Meyer-Lindenberg A, Schnell K, Opitz von Boberfeld C, Esslinger C, Kirsch P, et al. Brain function in carriers of a genome-wide supported bipolar disorder variant. Arch Gen Psychiatry. 2010;67(8):803–11. 10.1001/archgenpsychiatry.2010.94 20679588

[7] Fawcett J, Kravitz HM. Anxiety syndromes and their relationship to depressive illness. J Clin Psychiatry. 1983;44(8 Pt 2):8–11. 6874657

[8] Green EK, Grozeva D, Jones I, Jones L, Kirov G, Caesar S, et al. The bipolar disorder risk allele at CACNA1C also confers risk of recurrent major depression and of schizophrenia. Mol Psychiatry. 2010;15(10):1016–22. 10.1038/mp.2009.49 PMC3011210 19621016

[9] Grigoriadis S, Robinson GE. Gender issues in depression. Ann Clin Psychiatry. 2007;19(4):247–55. 10.1080/10401230701653294 18058282

[10] Hamilton M. A rating scale for depression. J Neurol Neurosurg Psychiatry. 1960;23:56–62. 10.1136/jnnp.23.1.56 PMC495331 14399272

[11] Hansen T, Olsen L, Lindow M, Jakobsen KD, Ullum H, Jonsson E, et al. Brain expressed microRNAs implicated in schizophrenia etiology. PLoS One. 2007;2(9):e873. 10.1371/journal.pone.0000873 PMC1964806 17849003

[12] Harrison PJ, Tunbridge EM, Dolphin AC, Hall J. Voltage-gated calcium channel blockers for psychiatric disorders: Genomic reappraisal. Br J Psychiatry. 2020;216(5):250–3. 10.1192/bjp.2019.157 PMC7557861 31230606

[13] Lavebratt C, Aberg E, Sjoholm LK, Forsell Y. Variations in FKBP5 and BDNF genes are suggestively associated with depression in a Swedish population-based cohort. J Affect Disord. 2010;125(1–3):249–55. 10.1016/j.jad.2010.02.113 20226536

[14] Lett TA, Zai CC, Tiwari AK, Shaikh SA, Likhodi O, Kennedy JL, et al. ANK3, CACNA1C and ZNF804A gene variants in bipolar disorders and psychosis subphenotype. World J Biol Psychiatry. 2011;12(5):392–7. 10.3109/15622975.2011.564655 21767209

[15] Mosheva M, Serretti A, Stukalin Y, Fabbri C, Hagin M, Horev S, et al. Association between CANCA1C gene rs1034936 polymorphism and alcohol dependence in bipolar disorder. J Affect Disord. 2020;261:181–6. 10.1016/j.jad.2019.10.015 31634677

[16] Odone A, Lugo A, Amerio A, Borroni E, Bosetti C, Carreras G, et al. COVID-19 lockdown impact on lifestyle habits of Italian adults. Acta Biomed. 2020;91(9-S):87–9. 10.23750/abm.v91i9-S.10122 PMC8023096 32701921

[17] Pasparakis E, Koiliari E, Zouraraki C, Tsapakis EM, Roussos P, Giakoumaki SG, et al. The effects of the CACNA1C rs1006737 A/G on affective startle modulation in healthy males. Eur Psychiatry. 2015;30(4):492–8. 10.1016/j.eurpsy.2015.03.004 25841664

[18] Pompili M, Serafini G, Innamorati M, Montebovi F, Palermo M, Campi S, et al. Car accidents as a method of suicide: a comprehensive overview. Forensic Sci Int. 2012;223(1–3):1–9. 10.1016/j.forsciint.2012.04.012 22576104

[19] Rao S, Yao Y, Zheng C, Ryan J, Mao C, Zhang F, et al. Common variants in CACNA1C and MDD susceptibility: A comprehensive meta-analysis. Am J Med Genet B Neuropsychiatr Genet. 2016;171(6):896–903. 10.1002/ajmg.b.32466 27260792

[20] Roussos P, Giakoumaki SG, Georgakopoulos A, Robakis NK, Bitsios P. The CACNA1C and ANK3 risk alleles impact on affective personality traits and startle reactivity but not on cognition or gating in healthy males. Bipolar Disord. 2011;13(3):250–9. 10.1111/j.1399-5618.2011.00924.x 21676128

[21] Sekiguchi F, Tsubota M, Kawabata A. Involvement of voltage-gated calcium channels in inflammation and inflammatory pain. Biol Pharm Bull. 2018;41(8):1127–34. 10.1248/bpb.b18-00054 30068860

[22] Serafini G, Parmigiani B, Amerio A, Aguglia A, Sher L, Amore M The psychological impact of COVID-19 on the mental health in the general population. QJM. 2020. 10.1093/qjmed/hcaa201 PMC7337855 32569360

[23] Strohmaier J, Amelang M, Hothorn LA, Witt SH, Nieratschker V, Gerhard D, et al. The psychiatric vulnerability gene CACNA1C and its sex-specific relationship with personality traits, resilience factors and depressive symptoms in the general population. Mol Psychiatry. 2013;18(5):607–13. 10.1038/mp.2012.53 22665259

[24] Troubat R, Barone P, Leman S, Desmidt T, Cressant A, Atanasova B, et al. Neuroinflammation and depression: A review. Eur J Neurosci. 2021;53(1):151–71. 10.1111/ejn.14720 32150310

[25] van Diermen L, van den Ameele S, Kamperman AM, Sabbe BCG, Vermeulen T, Schrijvers D, et al. Prediction of electroconvulsive therapy response and remission in major depression: Meta-analysis – CORRIGENDUM. Br J Psychiatry. 2018;212(5):322. 10.1192/bjp.2018.67 29609664

[26] Wray NR, Pergadia ML, Blackwood DH, Penninx BW, Gordon SD, Nyholt DR, et al. Genome-wide association study of major depressive disorder: New results, meta-analysis, and lessons learned. Mol Psychiatry. 2012;17(1):36–48. 10.1038/mp.2010.109 PMC3252611 21042317

